# Anticipating need for intensive care in the healthcare trajectory of patients with chronic disease: A qualitative study among specialists

**DOI:** 10.1371/journal.pone.0274936

**Published:** 2022-09-19

**Authors:** Alicia Taha, Marine Jacquier, Nicolas Meunier-Beillard, Fiona Ecarnot, Pascal Andreu, Jean-Baptiste Roudaut, Marie Labruyère, Jean-Philippe Rigaud, Jean-Pierre Quenot

**Affiliations:** 1 Service de Médecine Intensive-Réanimation, CHU Dijon-Bourgogne, Dijon, France; 2 Equipe Lipness, Centre de Recherche INSERM UMR1231 et LabEx LipSTIC, Université de Bourgogne-Franche Comté, Dijon, France; 3 INSERM, CIC 1432, Module Épidémiologie Clinique, Université de Bourgogne-Franche Comté, Dijon, France; 4 DRCI, USMR, CHU Dijon Bourgogne, Dijon, France; 5 EA3920, Department of Cardiology, University Hospital Besancon, Besançon, France; 6 Department of Intensive Care, Centre Hospitalier de Dieppe, Dieppe, France; 7 Espace de Réflexion Éthique de Normandie, University Hospital Caen, Caen, France; 8 Espace de Réflexion Éthique Bourgogne Franche-Comté (EREBFC), Dijon, France; Universitatsklinikum Regensburg, GERMANY

## Abstract

**Introduction:**

We investigated the reflections and perceptions of non-ICU physicians about anticipating the need for ICU admission in case of acute decompensation in patients with chronic disease.

**Methods:**

We performed a qualitative multicentre study using semi-structured interviews among non-ICU specialist physicians. The interview guide, developed in advance, focused on 3 questions: (1) What is your perception of ICU care? (2) How do you think advance directives can be integrated into the patient’s healthcare goals? and (3) How can the possibility of a need for ICU admission be integrated into the patient’s healthcare goals? Interviews were recorded, transcribed and analysed by thematic analysis. Interviews were performed until theoretical saturation was reached.

**Results:**

In total, 16 physicians (8 women, 8 men) were interviewed. The main themes related to intensive care being viewed as a distinct specialty, dispensing very technical care, and with major human and ethical challenges, especially regarding end-of-life issues. The participants also mentioned the difficulty in anticipating an acute decompensation, and the choices that might have to be made in such situations. The timing of discussions about potential decompensation of the patient, the medical culture and the presence of advance directives are issues that arise when attempting to anticipate the question of ICU admission in the patient’s healthcare goals or wishes.

**Conclusion:**

This study describes the perceptions that physicians treating patients with chronic disease have of intensive care, notably that it is a distinct and technical specialty that presents challenging medical and ethical situations. Our study also opens perspectives for actions that could promote a pluridisciplinary approach to anticipating acute decompensation and ICU requirements in patients with chronic disease.

## Introduction

Intensive care occupies a special role within the healthcare system, as it is a specialty that calls for substantial human and technical resources, for a relatively limited number of patients. The patients admitted to intensive care units (ICU) are among the sickest in the hospital, and have quite a specific profile [1, 2]. Indeed, Quenot et al [3] previously showed in an observational study including 1294 patients that 68% of patients admitted to the ICU requiring life support for failure of at least one organ, had at least one chronic disease. These patients often have more severe disease at admission, and have considerably higher mortality than patients without chronic disease [3–8]. Similarly, the life experiences of these patients prior to admission to ICU in terms of disability, incapacity or frailty [9] may partially explain the higher mortality, as well as the physical, psychological and cognitive repercussions observed after an ICU stay in patients with chronic disease, collectively termed “post-intensive care syndrome” (PICS) [10–13].

For several years now, there has been growing debate about the relevance of ICU admissions, in terms of the expected benefit for patients, and bearing in mind the moral duty to ensure distributive justice in the use of precious resources [14, 15]. These questions have come under particular scrutiny since the beginning of the SARS-CoV-2 epidemic [16, 17], which has placed unprecedented strain on the availability of ICU beds. The involvement of ICU physicians in care discussions before the need for ICU admission arises is therefore of paramount importance, as they are the specialists who are most acutely aware of the potential complications and serious socio-economic repercussions that a stay in the ICU may have for patients [18–21]. It is incumbent on ICU physicians to offer care that will provide the patient with a level of therapeutic engagement commensurate with that patient’s healthcare goals, personal desires and values, and with an acceptable anticipated quality of life after discharge from the ICU [22, 23]. However, this balance can be difficult to achieve, especially in emergency situations, with a trade-off often occurring between the risk of loss of opportunity if ICU admission is refused, and the risk of unreasonable therapeutic obstinacy if the patient’s stay in the ICU is ultimately non-beneficial [24, 25].

Thus, ideally, ICU physicians should be much more widely involved in decision-making before the occurrence of acute deteriorations requiring ICU admission, especially in patients with severe, unstable or progressing chronic diseases. This would help to anticipate the discussions about the need for, and relevance of admission to ICU. This is a thorny issue for several reasons: first, specialists often have difficulty making accurate prognostic estimations for their patients [26–28]; second, ICU physicians often lack information about the patient’s clinical state, prior history, previous quality of life or healthcare pathways [29]; third; it is difficult to broach the subject of acute deteriorations in health with patients and their families, as this often signals a turning point in the disease course [30]; and finally, there is a widespread lack of knowledge about the specific forms of care that can be provided in the ICU [22].

Against this background, we conducted semi-directed interviews to explore the perceptions of specialists caring for patients with chronic disease about the care delivered in the ICU; and to investigate their thoughts about the potential need for ICU admission in case of an acute deterioration in the patient’s health requiring life support therapy.

## Materials and methods

We performed a multicentre qualitative study using semi-directed interviews. Non-ICU specialist physicians caring for patients with chronic diseases in our institution were contacted by email and telephone. Trainees and students were not eligible for participation. We also excluded emergency room physicians, paediatricians, surgeons and all specialists working in the biological sciences (biochemistry, biology, toxicology, pharmacology). The participants were contacted by a researcher (AT) to organize an individual semi-structured interview at a convenient time. Reminders were sent to non-responders. Interviews were performed between April 2021 and January 2022 by a qualitative researcher (AT) and sociologist with experience in clinical research and ICU care (NMB). We developed an *ad hoc* interview guide using a methodology previously described elsewhere [31–33]. The interview guide focused on 3 main topics: (1) What perception of intensive care do specialists from other disciplines have? (2) How can the patients’ wishes and values be integrated into their healthcare goals? (3) How can the possible future need for ICU admission be integrated into the patient’s healthcare goals?

The interview guide was pilot tested with 3 physicians from our department; the findings from their interviews were not included in the analysis. The pilot interviews did not give rise to any major changes in the interview guide. The English translation of the interview guide is given in Table 1. The original French version is provided in S1 Table.

**Table 1:** English translation of the interview guide.

Themes	Sub-themes
Perception/knowledge of ICU	How do you define intensive care?
	(cues: type of patients, types of life support treatments)
	Do you previously do a rotation in ICU?
	Do you have any links/relations with the ICU?
Wishes / patient’s opinion / family	When is the right time to discuss the possibility of future ICU needs with patients (consultations? During acute phase of illness?)
	How can the topic be approached?
	What do you ask about (advance directives, ICU admission…?)
Advance Directives	Are they useful ?
	Are they common ?
Medical culture	Do you have personal experience of discussions about the level of therapeutic engagement?
	Contribution of other factors (religious beliefs…?)
Generation gap in anticipating care / level of therapeutic engagement	Rotations ?
	Culture ?

As with all qualitative interviews, the questions were open-ended and intended as a prompt to get the respondent to talk about the aspects that were most important to them, and to voice these concerns in their own words. Interviews were performed in a dedicated medical office or by phone. No other persons were present during the interview.

All interviews were recorded and transcribed for later analysis. Data were encoded to guarantee the anonymity of the participants. The corpus of discourse from all the interviews with physicians was analyzed using thematic analysis, as previously described by our group elsewhere [31–33]. In brief, interviews were coded independently by 2 of the coauthors (AT, NMB), the aim being to identify and categorize the different themes occurring in a cross-sectional manner across all interviews (i.e. topics common to several individuals). Each theme is then considered as a meaningful independent unit of discursive language. The different themes that arise during the interviews are recorded, taking into account major themes (significant points that are of major importance and well developed by the participants) and secondary themes (less well developed by the participants). The first level of analysis was performed individually by each researcher, then meetings were held to harmonize and decide on the major and secondary themes to be retained, and their regrouping into subject categories. Differences in interpretation were resolved by discussion and consensus. No software was used to assist with data management. Interviews were conducted until saturation was reached (i.e. the point at which new interviews failed to provide any new information about any of the points in the interview grid).

The analysis was validated by 4 authors (AT (reference author, female), NMB (male, sociologist, PhD), FE (female, clinical researcher, PhD), JPQ (male, critical care physician, MD, PhD). The final report was written by the same 4 authors, and approved by all. Results were not returned to the participants. Translation was performed after the results were finalized. Participants were informed that citations from their discourse may be used (translated into English) to illustrate the results of the study in a scientific publication, and all participants consented to this.

According to French legislation governing clinical research, this study did not require approval from an Ethics Committee, since no patients were involved. Consent was presumed by the fact that the participants agreed to be interviewed, but all participants were given an information leaflet and provided consent nevertheless.

## Results

A total of 28 physicians were contacted, of whom 16 agreed to be interviewed. After performing the 16 interviews with these 16 specialist physicians, point saturation was judged to have been reached in the data, and thus, no further interviews were performed and no further participants were sought. The average duration of the interviews was 36 minutes (range 20 to 60 minutes). The average age of participants was 36.9 years (range 30 to 52 years), and the average number (± standard deviation) of years’ experience in their discipline was 14 ±6.

The physicians were specialists in the following disciplines: internal medicine (n = 5), geriatrics (n = 3), nephrology (n = 3), pulmonology (n = 2), infectious diseases (n = 1), general medicine (n = 1), hepato-gastroenterology (n = 1).

Five major themes emerged from the interviews, namely: (1) Intensive care is seen as a specialty that is “separate” or “set apart”. (2) The timing of disease progression/deterioration and/or the stage of the chronic disease, as well as the medical culture in the wards surrounding end-of-life issues are factors that may be obstacles to discussions about anticipating need for ICU admission if an acute clinical situation were to arise. (3) Advance directives do not appear to be helpful in deciding about ICU admission. (4) Participants felt that training in palliative care and in ethical dilemmas, such as those encountered in end-of-life situations, is insufficient. (5) The idea of anticipating a potential need for ICU admission has been proposed numerous times, notably during the massive influx of patients during the SARS-CoV-2 epidemic, although this question is not systematically discussed in all Departments.

### 1. Intensive care: A distinct specialty

The majority of participants described intensive care as a specialty that is different from all the others, as it is highly technical and a source of anxiety, because of the permanent presence of death and dying.

“*Compared to other medical disciplines*, *I have this picture that’s both relatively technical*, *but also of an elite specialty*. *An elite specialty*, *with very technical care and major human and ethical dilemmas” (Geriatrician*, *male*, *36 years old)*

“*Maybe I have a negative view but for me*, *it’s demanding and difficult*, *in physical and mental terms*, *but also for family life” (Geriatrician*, *female*, *34 years old)*

Physicians who had done a rotation in an ICU during their medical studies had a better understanding of intensive care as a specialty, including the type of care that can be delivered in the ICU, the ethical aspects, and notably, the different levels of therapeutic engagement, which some physicians continue to apply in their daily practice in other disciplines:

“*Deciding on the level of therapeutic engagement is common practice in the ICU*, *and my rotation in intensive care opened my eyes to that*. *I still draw on that kind of thinking in my own specialty” (nephrologist*, *male*, *31 years old)*

“*I did a rotation in the ICU so I realize that “intensive care” doesn’t mean going to the maximum limit for every single patient*, *treating a patient for septic shock for 48 hours without necessarily going as far as dialysis or whatever*, *it’s sure that it’s easier when you have some idea*, *like at least 6 months*, *of what it’s like in intensive care” (Hepato-gastroenterologist*, *female*, *30 years old)*

A rotation in the ICU during medical school made it possible for these physicians to have an enhanced understanding of the consequences of (potentially long) ICU stays, and gave them a greater appreciation of the different levels of therapeutic engagement and the impact on patients’ quality of life.

“*Discussions about therapeutic engagement*, *especially in some chronic diseases*, *and decision-making…*. *It’s not always easy*. *Once you’ve worked in intensive care*, *you have a better understanding of what patients go through in the ICU” (Internal medicine*, *female*, *52 years old)*.

### 2. Anticipation a possible need for ICU admission

Some respondents reported that the close relationship they have with patients who have chronic disease sometimes makes it difficult for them to broach discussions about the level of therapeutic engagement, given the often subjective nature of views in this regard.

“*Patients that you see three times a week for dialysis*, *for example*, *well*, *talking to them about that seems complicated to me*. *Actually*, *I think I’d find it easier to talk about it to a patient that comes into the ICU during the night*, *a patient I don’t know” (nephrologist*, *female*, *30 years old)*

“*I think the physician who is following the patient in routine practice probably lacks objectivity and may not be the best placed to talk about ICU needs with that patient” (nephrologist*, *male*, *31 years old)*

The majority of respondents described their difficulties with accurate prognostication of acute deterioration, and also the difficulty they encounter trying to explain to their patients that during the course of their illness, there may be a need for ICU admission at some point along the management pathway.

“*Personally*, *I find it very difficult*, *to talk about how far to go*, *if the situation were to arise*, *like acute organ failure*, *there’s really only a very small minority of patients who are transferred to ICU in whom we could anticipate it” (Internal medicine physician*, *male*, *47 years old)*

The respondents also mentioned the utility of “nuanced” levels of therapeutic engagement that can be re-evaluated on a regular basis, as this may help certain physicians to come to a decision about the level of therapeutic engagement during hospitalisation.

“*It definitely makes things easier*, *but the problem with the level of therapeutic engagement is that it mustn’t be set in stone*, *you have to keep re-evaluating it all through the hospital stay*, *and the subtle differences in levels of engagement aren’t always well understood” (Internal medicine physician*, *male*, *40 years old)*

“*Putting a label on it can sometimes be off-putting*, *you should be able to have more nuanced levels*. *It doesn’t mean that you’re doing nothing*. *But because I know the consequences it can entail*, *I know that it can be off-putting to ask the question” (Internal medicine physician*, *female*, *34 years old)*

When the levels of therapeutic engagement are discussed, the best time appears to be during a hospital stay, when the physician can take some quality time with the patient to discuss it. Conversely, during a consultation, there is not enough time, and the question of a possible need for ICU care may not seem relevant, according to the participants in our study.

“*The vast majority of patients are fine*, *they come in as outpatients to the consultation*, *so it’d be more useful to discuss just the complex cases*, *and not every single case” Internal medicine physician*, *male*, *30 years old)*

“*But often the best time is when they’re in hospital*, *and you see them during your rounds every day*, *and can take the time to sit down beside them and talk*, *you can take more time than during consultations where you’re short of time” (Pulmonologist*, *female*, *40 years old)*

All the participants reported wide disparity in practices across medical specialties in terms of the level of care, which may be explained by differences in culture between wards, and by individual training, as some professionals may have more training in certain areas than others.

“*I think it’s a cultural thing among the physicians*, *the way each physician sees their profession*, *not to feel that they’re a hero*, *I think it’s very cultural*, *and related to education*. *It also depends on the specialties that you’ve worked in*, *and the experience you have of confronting those kinds of situations” (Internal medicine physician*, *female*, *52 years old)*

“*I really think that discussions about the level of therapeutic engagement depend on how you were trained*. *Every department or ward does its own thing*, *and young doctors eventually just start to think and act in the routine way of their department*. *I think that each specialty also sees life support of “its” organ as being less burdensome than life support in other disciplines” (Pulmonologist*, *male*, *31 years old)*

### 3. Advance directives: Are they really a useful tool?

All the respondents in our study concurred that advance directives are not widely known, and rarely written down. Even when advance directives are available, they are usually imprecise as regards the patient’s wishes, be it regarding the desired level of therapeutic engagement in case of ICU admission, or regarding their end-of-life wishes.

“*Advance directives are fine when they’re done right*, *and only when the patient knows what they’re talking about when they write them*, *because often they just write something like*, *“I don’t want you to go overboard trying to keep me alive”*, *but that’s not specific enough to be useful for making targeted decisions” (Geriatric medicine physician*, *male*, *34 years old)*

“*Personally*, *I’m not all that interested in advance directives because I have rarely had a patient give me a clear response*. *I get the impression that everyone just answers*, *“I don’t want unreasonable therapeutic obstinacy”*. *But nobody wants that*, *everyone agrees about that*.*” (Nephrologist*, *male*, *31 years old)*

The referring specialist or the general practitioner could have a key role to play in helping the patient to prepare advance directives, during a dedicated consultation.

“*Referring specialists or general practitioners could play a role in that*, *by telling the patient that if there are things you don’t want*, *then write down the list*, *and be specific” (Infectious diseases physician*, *female*, *38 years old)*

“*To make it easier*, *maybe there should be dedicated consultations with the general practitioner*, *with a specific billing code*, *to talk about it before the need arises*, *but in a context where they can take the time to discuss it” (Internal medicine physician*, *male*, *30 years old)*

Advance directives could be a useful tool for patients with chronic disease to explicitly and formally describe their wishes, but the directives need to be reviewed and revised regularly over time, according to the level of functional autonomy and cognitive function of the patient.

“*I find that the problem with advance directives is probably due to the progressive nature of the disease*. *The patient gradually becomes dependent*, *and in fact*, *patients recalibrate their level of tolerance*. *Something they wouldn’t have accepted 3 years before when they were in good form*, *well gradually they declined and lost autonomy but they’re still perfectly satisfied with their current state nonetheless” (Internal medicine physician*, *female*, *52 years old)*

“*Actually*, *as we age*, *we’re not as demanding as we were when we were younger*, *and your level of tolerance for disability is not the same as it was when you were young” (Internal medicine physician*, *female*, *34 years old)*

### 4. End-of-life and palliative care

Certain respondents in our study highlighted the existence of a “generation gap” in the implementation of palliative care in practice. The medical training received by younger physicians, better knowledge of end-of-life legislation and a shift towards more patient-centred care all contribute to making it easier for younger physicians to implement palliative care. Conversely, their relative lack of experience may make it hard for them to accept that a patient is at the end of life, with the ensuing decisional and attitudinal difficulties.

“*For sure*, *I think there’s a difference between younger and older physicians*. *The law changed in 2005 with the introduction of the Leonetti law*, *but not everyone is aware of those changes*. *We’re in a different paradigm now*, *even at societal level*, *we’re not in a paternalistic paradigm any more where the doctor decides about everything like we were God” (Internal medicine physician*, *male*, *30 years old)*

“*I think the older physicians we work with find it harder to stop curative therapy*. *They’re of a generation where they mostly never even asked that question” (Nephrologist*, *female*, *30 years)*

“*Sometimes when you’re young*, *it’s hard to have the necessary distance*, *sometimes it’s the younger doctors who tend to push harder” (Internal medicine physician*, *male*, *40 years old)*

The referring specialist rarely asks the patient about end-of-life wishes, and these are not often specified in the therapeutic goals. Geriatric medicine physicians are more accustomed to anticipating these issues with their patients.

“*Ah yes… I think we don’t like to let people die*, *because death is too scary” (Internal medicine physician*, *female*, *34 years old)*

“*It depends on the experience of the physician working in the ward*, *and their working habits*, *and maybe also how often they encounter death” (Pulmonologist*, *male*, *30 years old)*

“*Most of the time*, *geriatric patients have already thought about it*. *Really*, *asking them about their end-of-life wishes*, *it’s not disturbing*, *whereas asking someone of 60 years of age about their end-of-life wishes…*..*” (Geriatric medicine physician*, *female*, *34 years old)*

The question of the end-of-life seems to be difficult to address, because death is often perceived as a failure by doctors:

“*It’s always the same thing*, *the problem of death*, *and telling people “I can’t do anything more for you”*, *it’s hard*. *But in reality*, *it’s not a case of “I can’t do anything more for you”*, *but rather “you have a disease that we don’t know how to treat” and I think that’s a failure for the doctor*, *yes” (Internal medicine physician*, *female*, *34 years old)*

“*I think that doctors can’t let go*, *we always go beyond” (general medicine physician*, *female*, *52 years old)*

“*I think we’re all different*, *and we can or can’t accept the fact that we can’t save everyone” (Internal medicine physician*, *female*, *52 years old)*

### 5. Prioritising access to care during the SARS CoV-2 pandemic

The need to anticipate possible ICU admission or non-admission became acutely salient during the SARS-CoV-2 pandemic because of the unprecedented strain on ICU bed availability, with insufficient beds to cater for the huge influx of critically ill patients. Conversely, the question was not previously discussed systematically for all patients hospitalized, regardless of the disease requiring intensive care.

“*The COVID crisis really brought to a head this whole question about ICU admission*. *The decision is made systematically now as soon as they are admitted to the ward” (Nephrologist*, *male*, *31 years old)*

## Discussion

In the healthcare trajectory of patients with chronic disease, close collaboration between specialists and ICU physicians is essential to guide decisions about the need for ICU care [34, 35]. The former know the patient well, while the latter know the forms of care available in the ICU that might be useful for temporary life support. Both roles are complementary and necessary to deliver care that is in line with the patient’s pre-established healthcare goals, and the patient’s wishes and values [36]. In our study, the key points to emerge from the interviews with non-ICU specialists were the following: First, intensive care is seen as a very distinct specialty that is “worrying” because of the pervasive presence of death. Second, the timing of disease progression in chronic illness, and the medical culture that reigns in some departments regarding end-of-life issues may be obstacles to discussions about anticipating future ICU needs in case of acute deterioration. Third, advance directives are not generally considered useful in deciding on whether or not to admit a patient to ICU. Fourth, education and training in palliative care and end-of-life ethics appear to be insufficient. Finally, there was heightened awareness of the need to anticipate ICU admissions during the SARS-CoV-2 pandemic.

In a recent qualitative study using individual in-depth interviews, Cullati et al [37] explored the respective roles of internists and intensivists in ICU admission decisions. They identified two main roles, namely practical clinical roles and identity roles, which contribute to the doctors’ professional identity. In our study, intensive care was perceived as a “separate” or “distinct” specialty, set apart from other medical disciplines by its highly technical care. Nevertheless, the participants all acknowledge the professional stature of ICU physicians when it comes to making decisions, and discussing complex situations that could lead to end-of-life decisions. Furthermore, previous experience of working in the intensive care environment, notably thanks to having done rotations in the ICU during medical training, is helpful for young doctors. It gives them a better understanding of the types of emergencies that can arise both within, and out of intensive care, as well as the care that can be offered in the ICU. It also raises their awareness of the ethical dilemmas that caregivers may face during the healthcare pathway of patients in the ICU, especially those at the end of life [38].

In France, the so-called “Leonetti law” [39], introduced in 2005 and relating to the rights of patients and the end-of-life, lays down the legal framework for withholding and withdrawing care at the end-of-life. The principles of collegiality and multidisciplinarity in end-of-life discussions are enshrined in this law with a view to keeping subjectivity in check. This subjectivity was referred to by the specialists in our study, who also spoke of their difficulty, not to say fear of broaching the subject of acute clinical deterioration with a patient with a view to prompting reflection about the desired level of therapeutic engagement. This dilemma is similar to that encountered by the ICU physician with the patient and family when there have been no discussions or decisions made before the need for ICU admission arises. Indeed, the ICU physician is then in the awkward position of choosing between loss-of-opportunity for the patient if admission is refused, or alternatively, delivery of care that may later be deemed disproportionately burdensome [40, 41] or even futile [42]. This can result in ICU admissions that are judged to have been non-beneficial [25]. The findings of this study plead in favour of enhanced collaboration between intensivists and referring specialists when there is debate about a potential ICU admission, especially if the question is being considered before any acute organ failure has occurred [43, 44]. Several reasons can be proposed to explain this [45]. First, intensivists are best placed to judge which types of intensive care treatments can be proposed to the patient. Second, they are also best placed to assess the patient’s prognosis, based on the presence or absence of organ failure. Third, they are also the best placed to explain intensive care to patients and their families, as well as its possible limitations and consequences. Conversely, the referring specialist is best placed to inform the intensivists about the patient’s wishes and healthcare goals, which are essential foundations to be considered during the ICU stay.

Based on the findings of our study, new perspectives emerge for improving the healthcare pathways of patients with chronic disease. To this day, some patient files still contain the notation “not to be resuscitated”, sometimes even without consulting the patient. Such injunctions carry heavy consequences, and imply that no resuscitation is to be attempted in the patient in the event of an acute deterioration. On the contrary, the absence of such a notation implies that any and all resuscitation efforts should be attempted, often without there having been any discussion of the question with the patient in advance. The participants in our study highlighted these discrepancies and the need for a holistic, global approach to the question. In ethical terms, arbitrary and impenetrable categorisation must be replaced by collective, pluridisciplinary discussion of the level of therapeutic engagement, and this process must be engaged together with the patient and family, commensurate with the patient’s wishes and healthcare goals. The different possible levels of therapeutic engagement were mentioned by our respondents, who considered them to be part of the intensivists’ role [38]. This concept is now widely known, and contributes to improving the quality of management of patients at the end-of-life [46, 47], and reducing suffering among caregivers [48].

There is strong ethical component to the discussions about admission decisions to ICU, which is coherent with the recently published recommendations from the Task Force of World Federation of Societies of Intensive and Critical Care Medicine [22, 49], and the more recent French guidelines for admission and management of critical care patients in a pandemic context [16]. In light of these recommendations, in going beyond their usual sphere of activity to take on the role of consultant [29, 43] and by aiding the definition of suitable levels of therapeutic engagement for patients in the wards before a need for ICU care arises, ICU physicians have become a central actor in the organisation of the healthcare trajectory and healthcare goals [15, 35]. This role is even more salient during the current pandemic, when ICU beds become a rare and precious resource.

Advance care planning (ACP) has been gaining traction in a number of countries (e.g. United Kingdom, USA, Australia…) for many years [50]. This process complements the definition of levels of therapeutic engagement and is a dynamic process involving the patient, family and healthcare providers, and reviewed regularly, to define common goals regarding future medical therapy. The overarching aim is to ensure the patient receives care that is consistent with their personal preferences and values. ACP is a proactive and anticipatory process that is designed to facilitate decision-making when emergency situations arise, or in cases where the patient is no longer able to express their own preferences. It could easily encompass the preparation of advance directives, if the patient so desires. In addition to respecting the patient’s preferences, ACP has other advantages, notably for patients with chronic disease [51–53], including an increased number of patients with advance directives (and the reflection that goes with their preparation), improved alignment of care with patient wishes, and enhanced communication between the patient, family and healthcare professionals [15].

Conversely, there are numerous obstacles that may hamper successful ACP, including the timing of ACP with regard to disease progression, the training of the physicians caring for the patient, the difficulty for patients to confront the end-of-life and death, and the patients’ lack of knowledge about the progressive nature of their disease [51, 54]. These factors were also highlighted by the participants in our study.

A further perspective that could improve patient management is the implementation of dedicated post-ICU consultations [55]. Indeed, it has been reported in the literature that patients may suffer physical, psychological and cognitive symptoms after a stay in the ICU, collectively termed post-intensive care syndrome (PICS) [56]. Following-up on patients to see how they fare after discharge from the ICU could be useful to evaluate the quality of the ICU management, patients’ outcomes, control of the disease that led to ICU admission, and the progression (if any) of the chronic disease [57, 58]. During post-ICU follow-up, attention should also be paid to the family, who may also suffer consequences relating to the ICU admission of a loved one [59]. Finally, it is also an ideal opportunity for ethical reflection on the question of whether or not to readmit the patient to the ICU, should the need arise [31, 34], discussion of the patient’s wishes for end-of-life care, including the writing of advance directives.

### Study limitations

This study has some limitations. Firstly, as with all qualitative studies, there may be potential for social desirability bias in the responses, particularly when asked about ethics issues. However, the potential for such bias was minimized by asking participants to describe concrete examples of situations they had personally experienced. Secondly, the interview guide did not cover the full spectrum of possible questions and topics raised by the anticipation of ICU admission. We chose to limit our scope to patients with chronic diseases. Therefore, the results of this study may not be generalizable to other patient groups likely to need ICU admission. Finally, although the number of participants may appear relatively small, it is in line with the standards of qualitative research, and was sufficient to reach saturation in this study [31–34]. A strength of the study is the representativeness of the different medical specialties outside of the ICU, which confers greater generalizability on our findings.

## Conclusion

This study highlights the perceptions of intensive care held by physicians caring for patients with chronic diseases, namely that intensive care is a distinct and highly technical specialty, where medical and ethical challenges are encountered. In view of these perceptions, it is important to anticipate the need for ICU admission, especially in patients with chronic illness whose disease course may be punctuated by acute episodes of deterioration, in ordert to ensure that ICU admission is warranted and beneficial for the patient. The anticipation of the need for ICU care should jointly involve the referring specialist and ICU physicians, and should be in line with the patient’s wishes and healthcare goals.

## Supporting information

S1 Table Original French version of the interview guide. (DOCX)
