# Supplementary material for: Anticipating need for intensive care in the healthcare trajectory of patients with chronic disease: A qualitative study among specialists
Source: PLoS One. 2022 Sep 19;17(9):e0274936. doi: 10.1371/journal.pone.0274936 (PMC9484637; doi:10.1371/journal.pone.0274936)
Supplement: S1 Table — (DOCX) [file pone.0274936.s001.docx]

**Supplementary Table S1 : Original French version of the Interview Guide**

Guide d’entretien

| Thèmes | Sous thèmes |
| --- | --- |
| Perception/connaissance de la réanimation | Quelle est votre définition de la réanimation (types de patients, types de traitement de suppléance), stage en internat ? lien avec ces services ? |
| Souhait/avis du patient/proche | Comment demander ? (Facilité à poser la question, être à l’aise avec le thème). Quelle discussion ? Quelle forme ? (RCP, DA…) Quel moment ? (Consultation, phase aiguë) |
| Directives anticipées | Utile ? fréquent ? |
| Culture médicale | Expérience personnelle sur les discussions de niveau de soin ? Religion ? |
| Différence générationnelle sur anticipation de soins/niveaux de soins | Stages ? Culture ? |
